# Supplementary figures and images for: α-Lipoic acid prevents against cisplatin cytotoxicity via activation of the NRF2/HO-1 antioxidant pathway
Source: PLoS One. 2019 Dec 26;14(12):e0226769. doi: 10.1371/journal.pone.0226769 (PMC6932784; doi:10.1371/journal.pone.0226769)

|            |   |   |   |   |
|------------|---|---|---|---|
| <i>ALA</i> | - | + | - | + |
| <i>Cis</i> | - | - | + | + |

|            |   |   |   |   |
|------------|---|---|---|---|
| <i>ALA</i> | - | + | - | + |
| <i>Cis</i> | - | - | + | + |

|            |   |   |   |   |
|------------|---|---|---|---|
| <i>ALA</i> | - | + | - | + |
| <i>Cis</i> | - | - | + | + |

|            |   |   |   |   |
|------------|---|---|---|---|
| <i>ALA</i> | - | + | - | + |
| <i>Cis</i> | - | - | + | + |

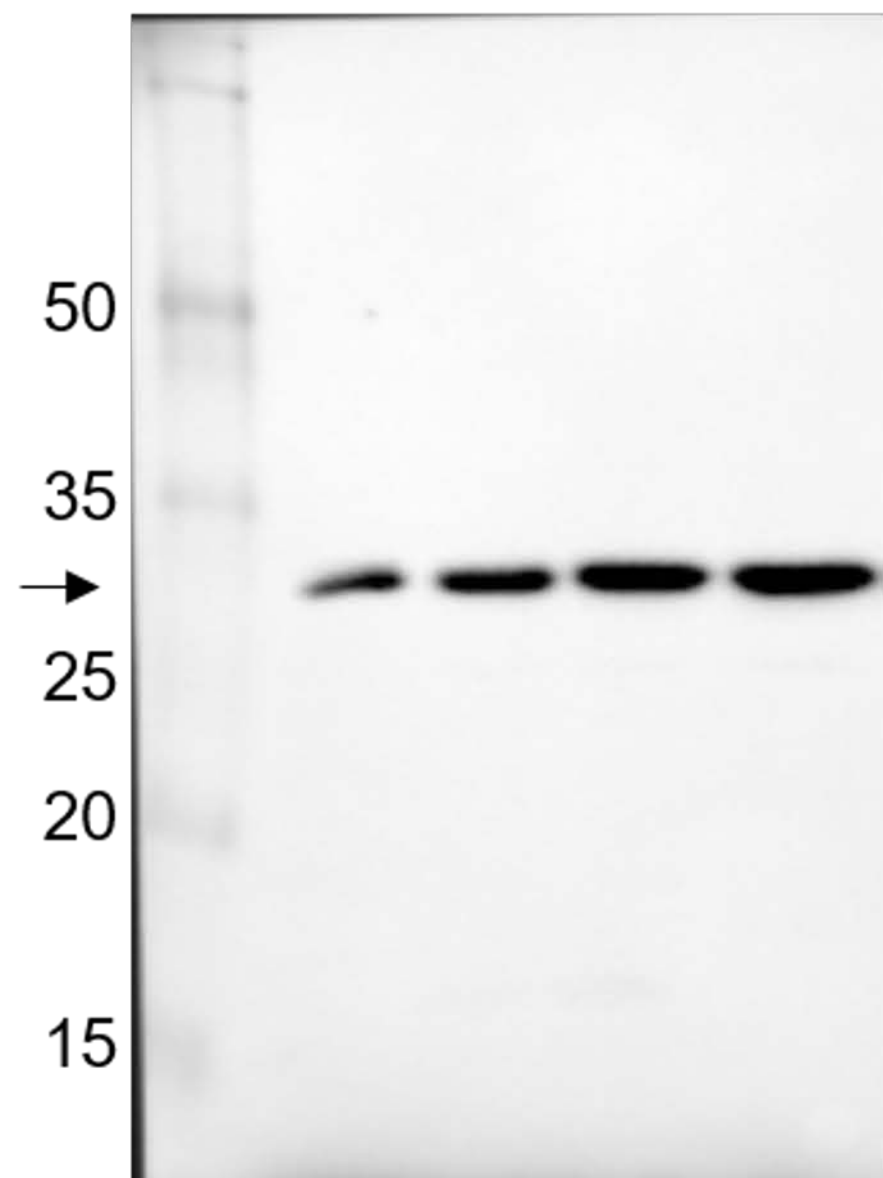

HO1

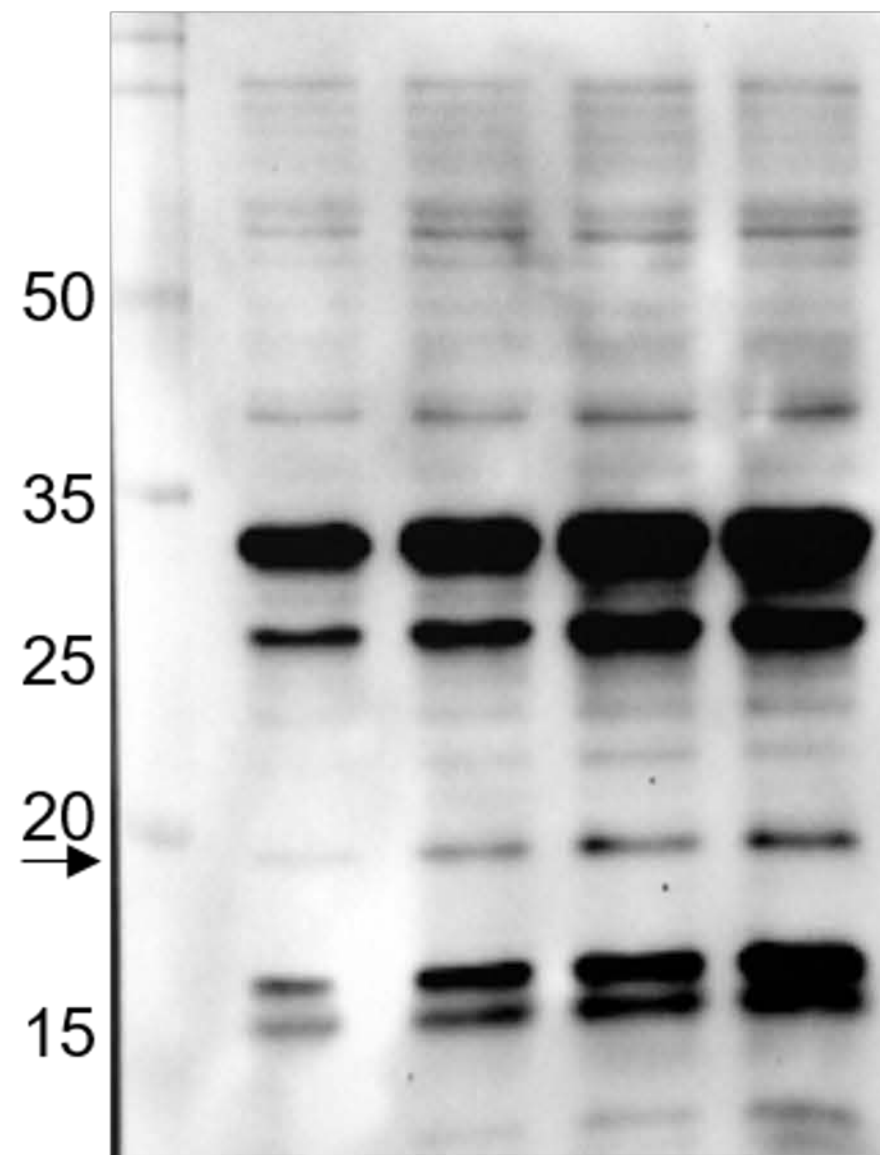

SOD1

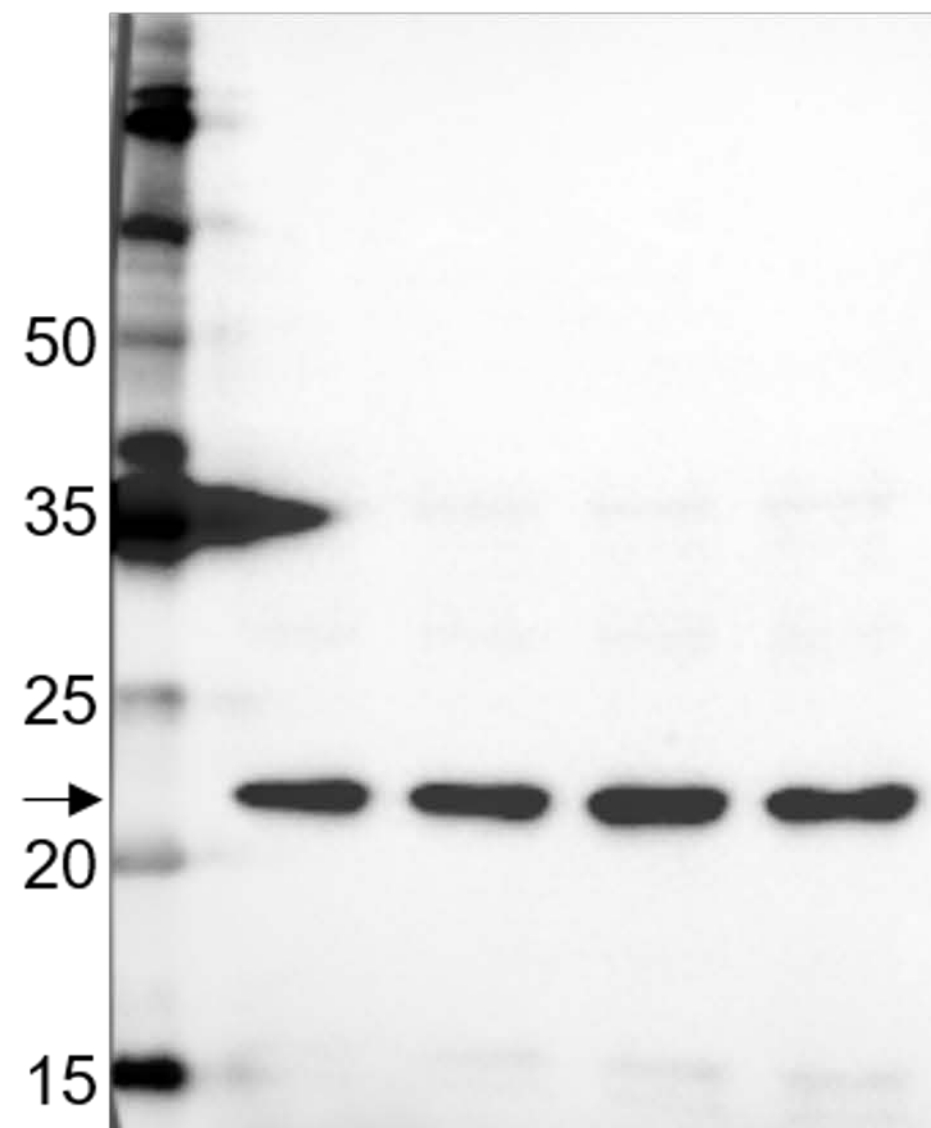

SOD2

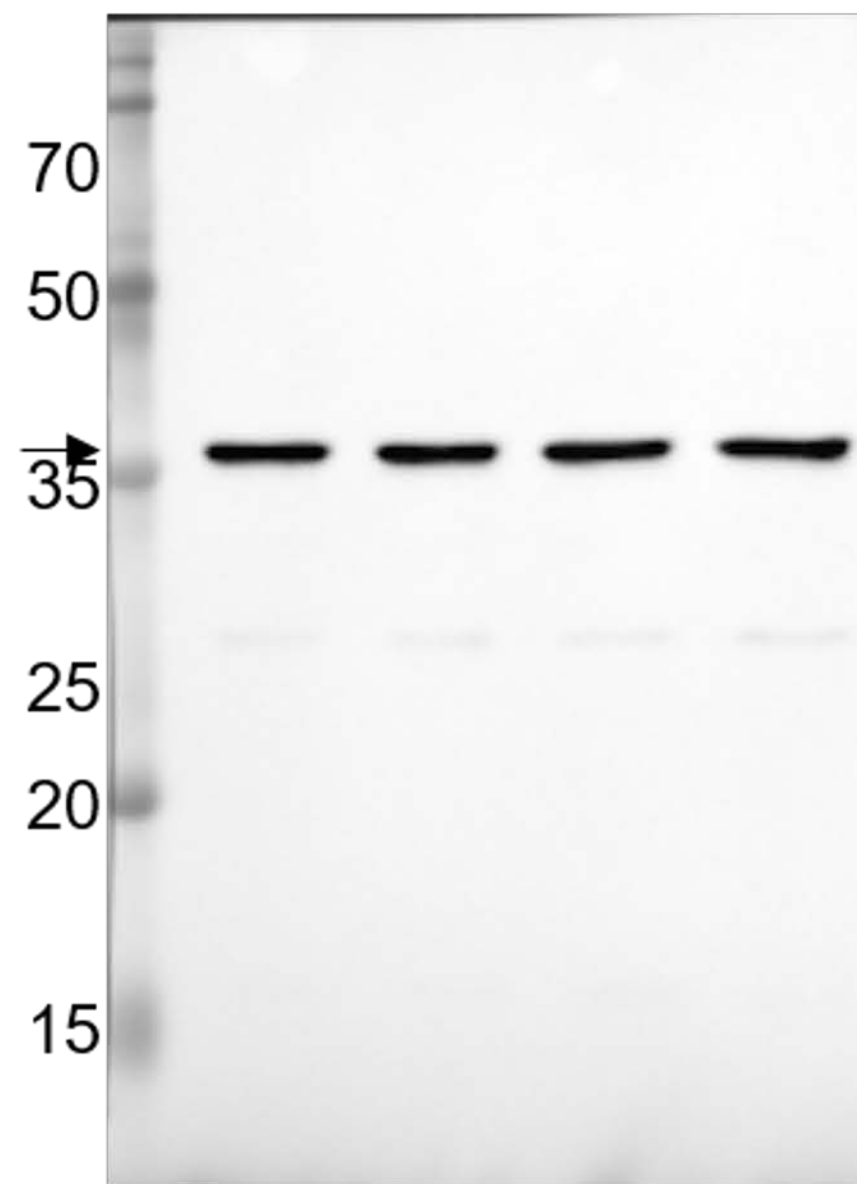

GAPDH

Supplement: S1 Fig — (PDF) [file pone.0226769.s001.pdf]

|               |   |   |   |   |   |   |
|---------------|---|---|---|---|---|---|
| <i>LA</i>     | - | + | - | - | + | + |
| <i>siNrf2</i> | - | - | + | - | - | + |
| <i>Cis</i>    | - | - | - | + | + | + |

|               |   |   |   |   |   |   |
|---------------|---|---|---|---|---|---|
| <i>LA</i>     | - | + | - | - | + | + |
| <i>siNrf2</i> | - | - | + | - | - | + |
| <i>Cis</i>    | - | - | - | + | + | + |

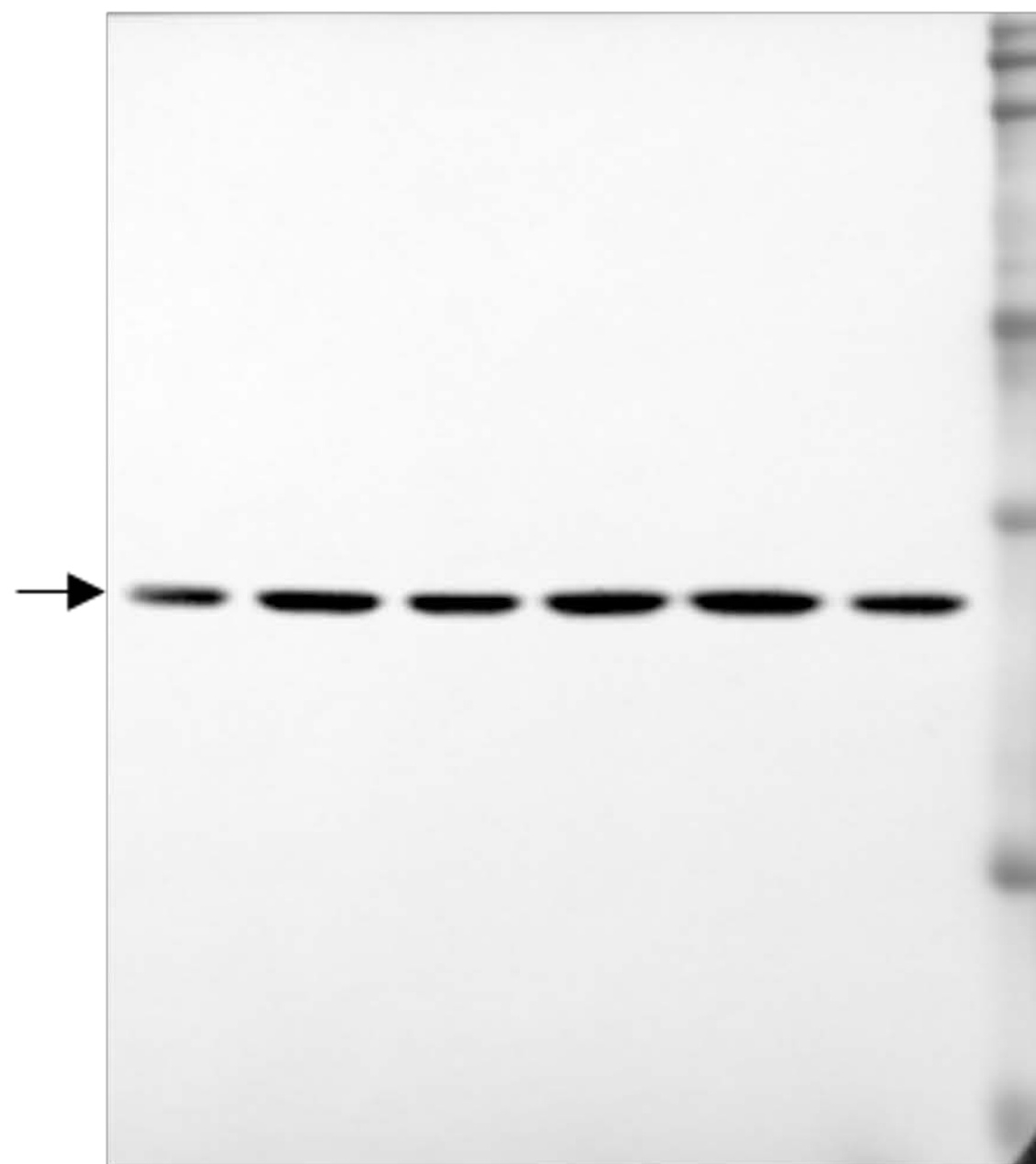

HO-1

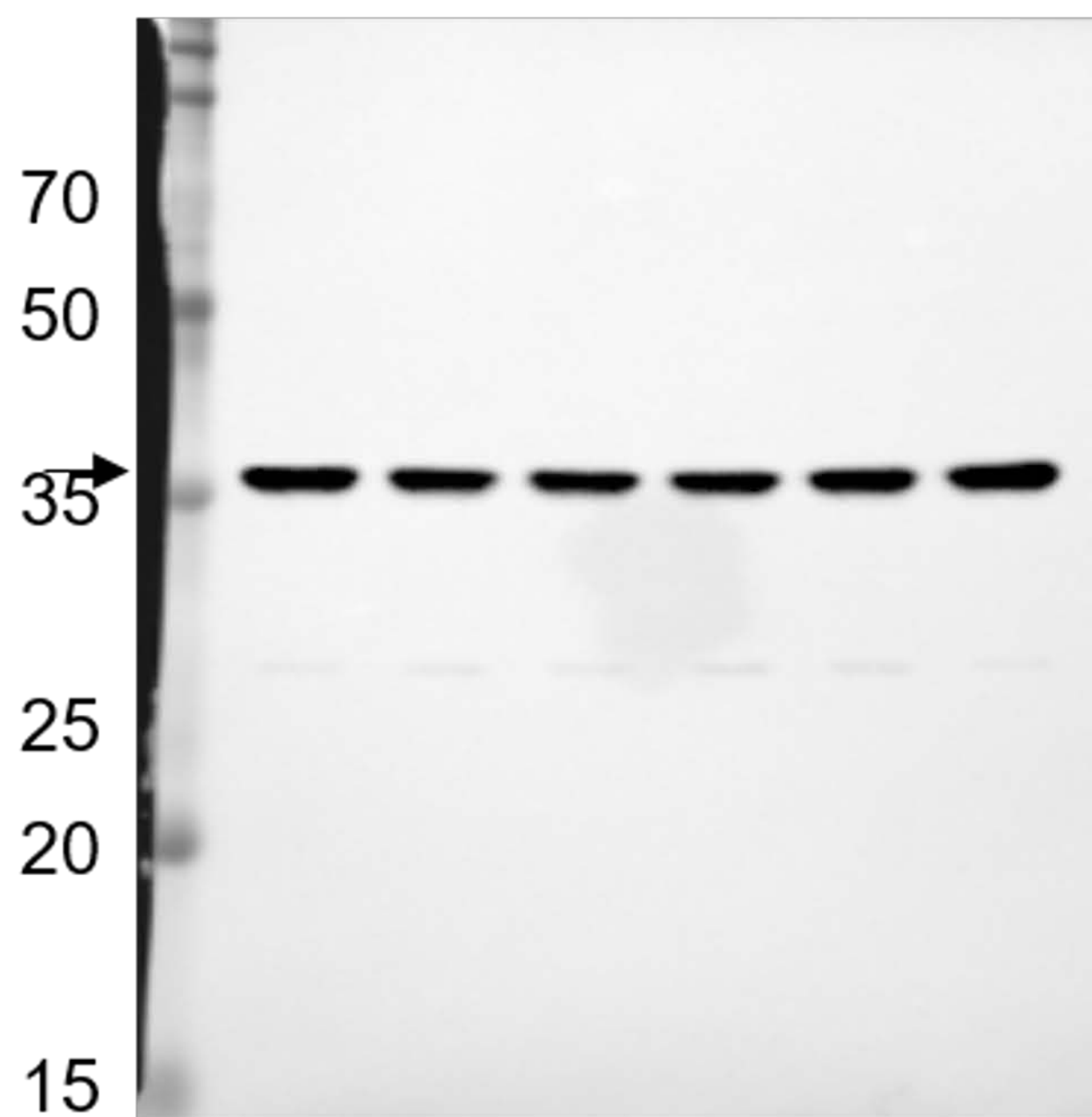

GAPDH

Supplement: S3 Fig — (PDF) [file pone.0226769.s003.pdf]

|               |   |   |   |   |   |
|---------------|---|---|---|---|---|
| <i>LA</i>     | - | + | - | + | + |
| <i>siNrf2</i> | - | - | - | + | + |
| <i>Cis</i>    | - | - | + | - | + |

|               |   |   |   |   |   |
|---------------|---|---|---|---|---|
| <i>LA</i>     | - | + | - | + | + |
| <i>siNrf2</i> | - | - | - | + | + |
| <i>Cis</i>    | - | - | + | - | + |

|               |   |   |   |   |   |
|---------------|---|---|---|---|---|
| <i>LA</i>     | - | + | - | + | + |
| <i>siNrf2</i> | - | - | - | + | + |
| <i>Cis</i>    | - | - | + | - | + |

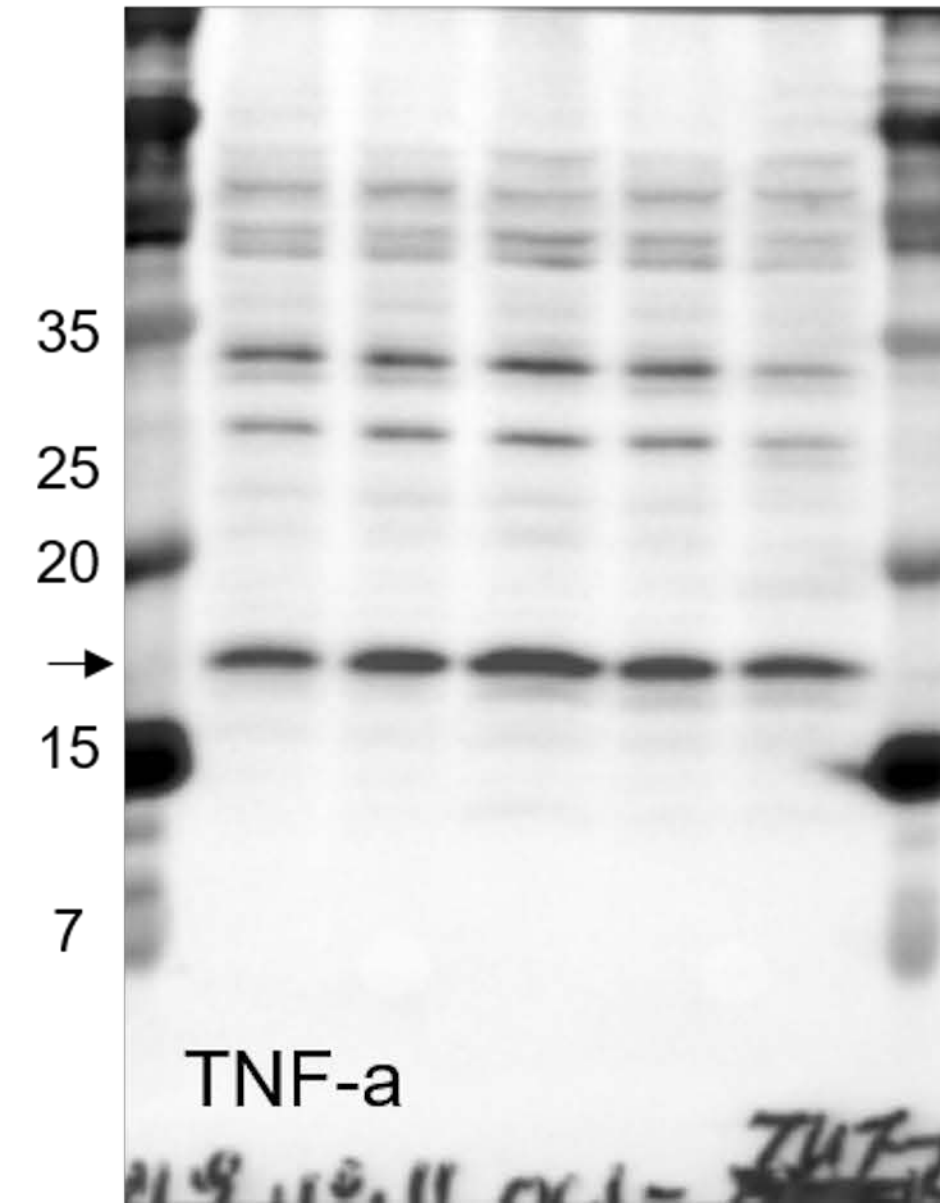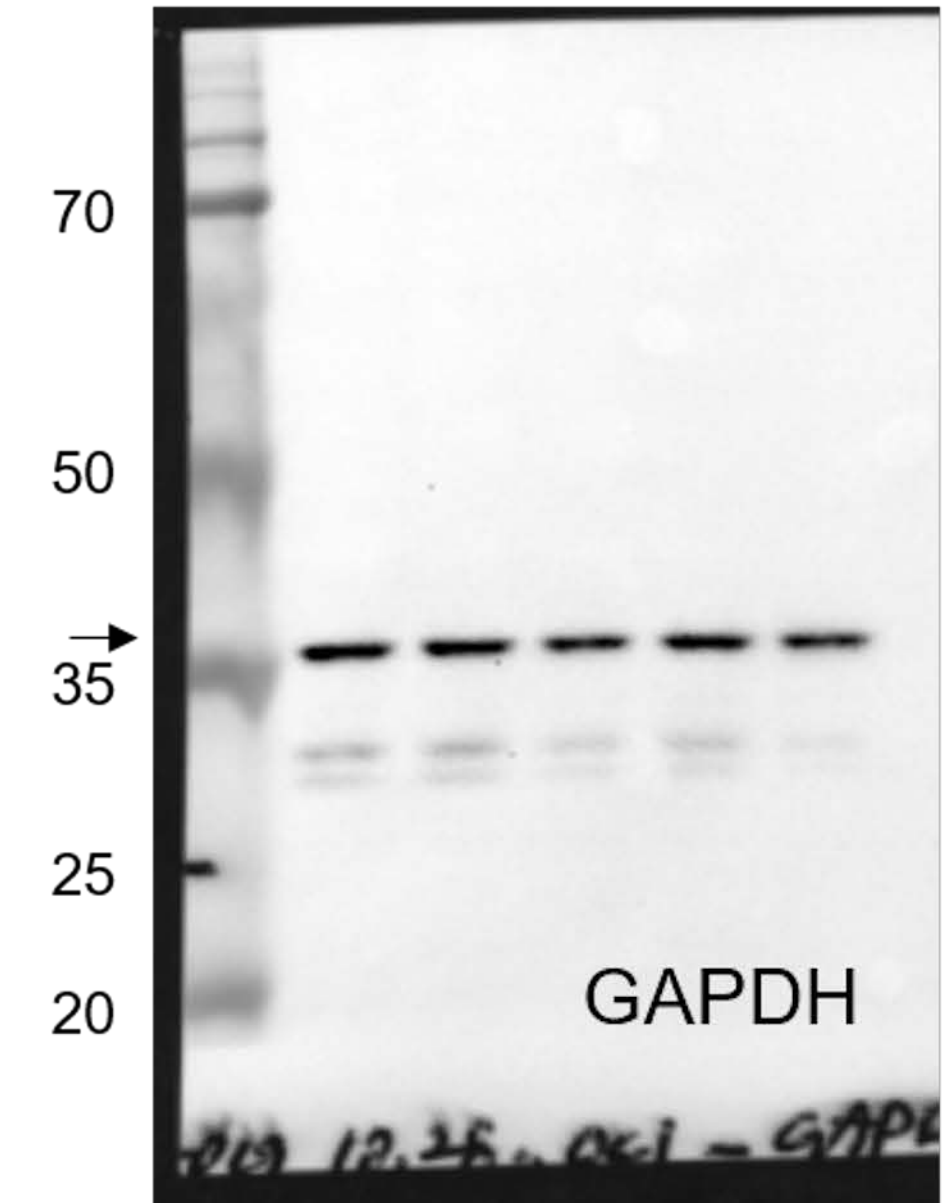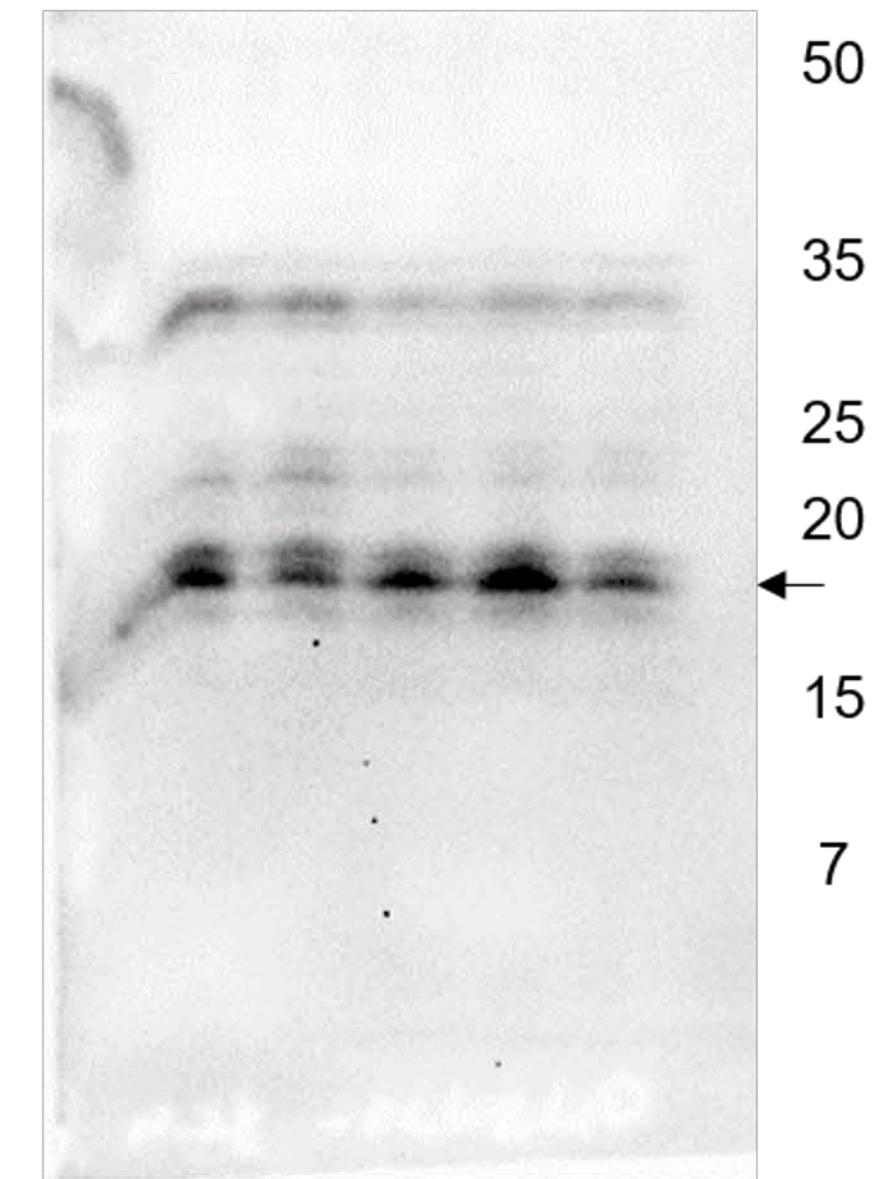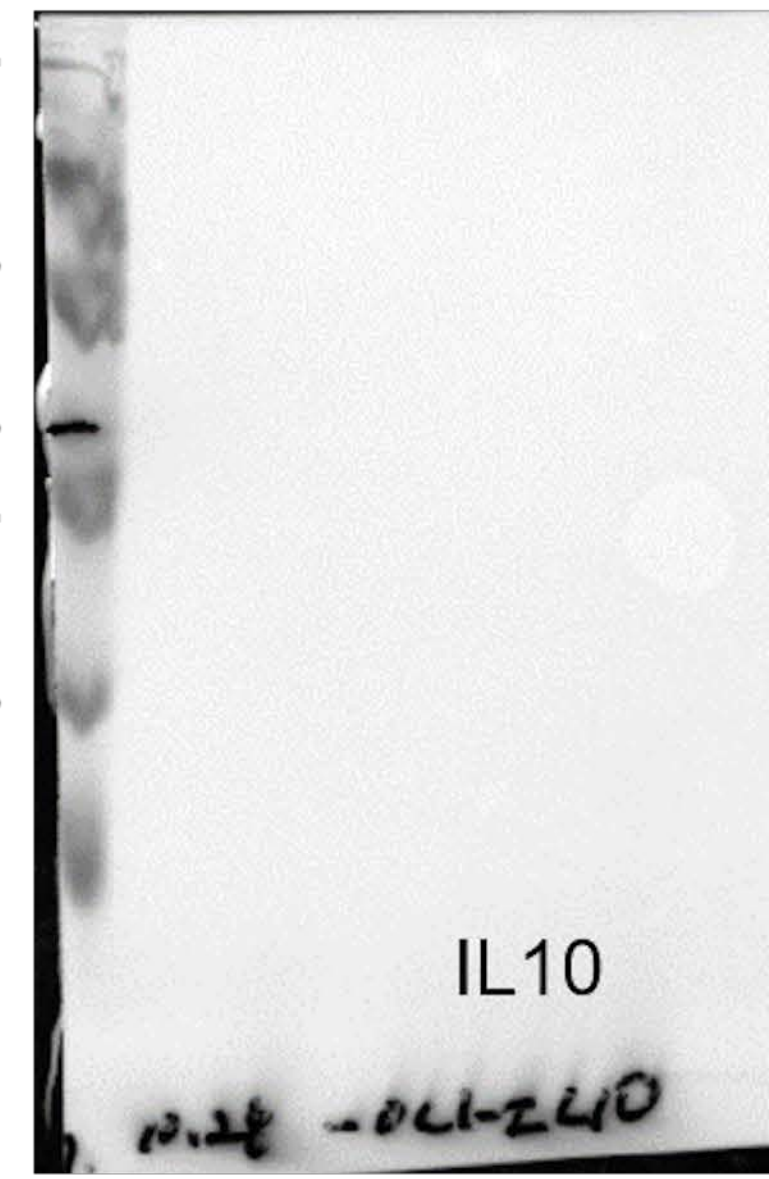

Supplement: S4 Fig — (PDF) [file pone.0226769.s004.pdf]
