# Supplementary material for: α-Lipoic acid prevents against cisplatin cytotoxicity via activation of the NRF2/HO-1 antioxidant pathway
Source: PLoS One. 2019 Dec 26;14(12):e0226769. doi: 10.1371/journal.pone.0226769 (PMC6932784; doi:10.1371/journal.pone.0226769)
Supplement: S2 Fig — (PDF) [file pone.0226769.s002.pdf]

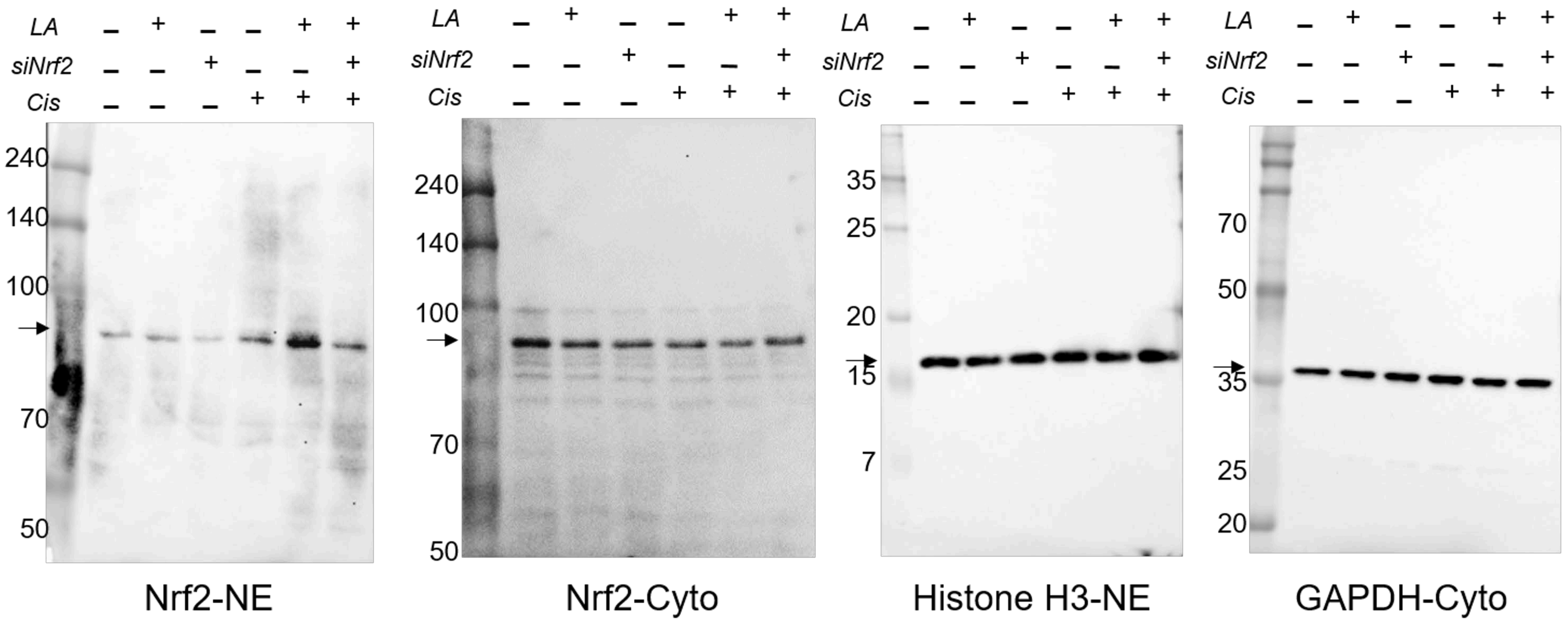

LA
siNrf2
Cis
240
140
100
70
50
35
25
20
15
7
Nrf2-NE
Nrf2-Cyto
Histone H3-NE
GAPDH-Cyto
